# First Case of Cutaneous Coinfection with *Aspergillus flavus* and *Klebsiella pneumoniae*: Case Report and Literature Review

**DOI:** 10.3390/diagnostics16020183

**Published:** 2026-01-07

**Authors:** Simona Maria Borta, Zsolt Gyori, Cosmin Catalin Bacean, Romana Olivia Popetiu, Cristina Petrine, Melani Zarici, Lavinia Palaghian, Adrian Silviu Crisan

**Affiliations:** 1 Department of Internal Medicine, Faculty of Medicine, “Vasile Goldiș” Western University of Arad, Bulevardul Revoluției 94, 310025 Arad, Romania; popetiur@gmail.com; 2 Arad County Clinical Emergency Clinical Hospital, Str. Andrényi Károly Nr. 2-4, 310037 Arad, Romania; dr.gyorizsolt.med@gmail.com (Z.G.); bacean_cosmin@yahoo.com (C.C.B.); petrinecristina@gmail.com (C.P.); 3 Department of Microsurgery and Reconstructive Surgery, Faculty of Medicine, “Vasile Goldiș” Western University of Arad, Bulevardul Revoluției 94, 310025 Arad, Romania; 4 “Pius Branzei” Timisoara County Clinical Emergency Hospital, Str. Bulevardul Liviu Rebreanu, Nr. 156, 300723 Timisoara, Romania; melanizarici28@icloud.com; 5 “Victor Popescu” Timisoara Military Clinical Emergency Hospital, Str. Gheorghe Lazar, Nr. 7, 300723 Timisoara, Romania; lpalaghian@yahoo.com; 6 Department of Critical Care and Emergency Medicine, “Vasile Goldiș” Western University of Arad, Bulevardul Revoluției 94, 310025 Arad, Romania

**Keywords:** aspergillosis, *Klebsiella pneumoniae*, coinfection, immunocompetence

## Abstract

**Background and Clinical Significance:** Cutaneous aspergillosis caused by *Aspergillus flavus* is rare and coinfection with *Klebsiella pneumoniae* was reported only in pulmonary disease. **Case Presentation:** We describe a 57-year-old woman with no prior comorbidities who developed septic shock requiring intensive care, broad-spectrum antibiotics, corticosteroids, and renal replacement therapy. Six days after discharge, she was readmitted with fever, leukopenia, thrombocytopenia, cavitary lung lesions, and multiple erythematous nodules on the limbs and mammary regions. Bronchial aspirate cultures detected *K. pneumoniae*, while progressive cutaneous lesions required surgical debridement. Histopathology revealed angioinvasive septate hyphae, and MALDI-TOF identified *A. flavus*. The *K. pneumoniae* strain was extensively drug resistant; *A. flavus* was susceptible only to azoles. Despite targeted therapy, lesions progressed requiring bilateral mastectomy. **Conclusions:** This case illustrates a previously unreported scenario in which secondary immunosuppression after severe sepsis led to concurrent cutaneous *A. flavus* infection and extensively drug-resistant (XDR) *K. pneumoniae*. Early recognition of mixed fungal–bacterial infections is essential for appropriate management.

## 1. Introduction

Driven by a complex interplay between multiple factors (e.g., expansion of intensive care medicine, broad-spectrum antibiotic overuse, rising fungal resistance, aging population), invasive fungal infections emerged as a major yet neglected cause of mortality and morbidity over the past three decades [1]. The profile of affected patients expanded from high-risk individuals, primarily cases with neoplasia, diabetes mellitus, acquired immunodeficiency syndrome (AIDS), and organ transplantation, to critically ill pediatric and adult patients [1]. A ubiquitous saprotrophic and pathogenic fungus, *Aspergillus flavus* (Link, 1809) is the second most common *Aspergillus* species worldwide and the main etiological factor behind cutaneous aspergillosis [2,3]. This infection characterized by polymorphic cutaneous lesions (e.g., macules, papules, plaques, ulcerations with central necrosis, pustules) is regarded as less dangerous than other forms of aspergillosis although coinfections with other fungi or bacteria may be associated with rapid progression, aggressive clinical presentation, more complicated treatment, and worse prognosis [4]. This may be the case of coinfection with *Klebsiella pneumonia*—an important Gram-negative, nonmotile pathogen that is responsible for a variety of common infections, including urinary tract infections, pneumonia, bacteremia, and skin and liver abscesses. This bacteria has become a major global health problem due to the rapid spread of extensively drug-resistant (XDR) forms and the worldwide spread of the hypervirulent type [5].

## 2. Case Presentation

We present the case of a 57-year-old women with no remarkable medical history, who was admitted to the intensive care unit (ICU) of the Arad County Emergency Clinical Hospital (SCJU Arad). No previous hospitalizations were documented in the patient’s electronic medical record, and the patient had no known comorbidities registered with the family physician. The patient reported a severely compromised general condition, febrile syndrome, loss of appetite, hypotension, marked fatigue, and anuria, with symptoms debuting approximately two days pre-admission. On admission, she was conscious and cooperative, but hemodynamically unstable, with a mild hypoxemia (SpO_2_ = 92%), tachycardia (120 beats per minute), low blood pressure (80/40 mm Hg), marbled skin, anuria, severe leukocytosis, severe systemic inflammatory response (SIRS), elevated blood urea nitrogen (BUN), dyselectrolytemia, and loaded urinary sediment. Fluid and electrolyte correction therapy was initiated, together with empirical antibiotic therapy with third generation cephalosporins, macrolides, corticosteroid therapy, vasopressor support, hepatoprotectors, proton pump inhibitors (PPIs), and anticoagulants in prophylactic doses. Urinary, pharyngeal, nasal, and blood cultures were also collected.

After three days of treatment, the clinical and biological parameters and persistent anuria required the initiation of renal replacement therapy via hemofiltration. A positive culture with *Staphylococcus epidermidis* was detected, with the antibiotic regimen being adjusted according to the antibiogram results. To ensure accurate and reliable bacterial detection, analysis was conducted in paired blood culture samples collected at a 1 h interval. Both samples yielded identical antimicrobial susceptibility profiles, showing the presence of *S. epidermidis*. This bacterium was susceptible to erythromycin, vancomycin, and tigecycline, but resistant to oxacillin, gentamicin, fluoroquinolones, clindamycin, linezolid, teicoplanin, tetracycline, fusidic acid, rifampicin, and trimethoprim–sulfamethoxazole. The complete antibiogram is shown in Table 1. The clinical course was favorable, the biological parameters improved with systemic inflammation biomarkers (e.g., C-reactive protein, erythrocyte sedimentation rate, fibrinogen) reaching normal values, BUN levels decreased, and diuresis was normal. As a result, the patient was discharged after 17 days of hospitalization in the ICU.

**Figure 1:**
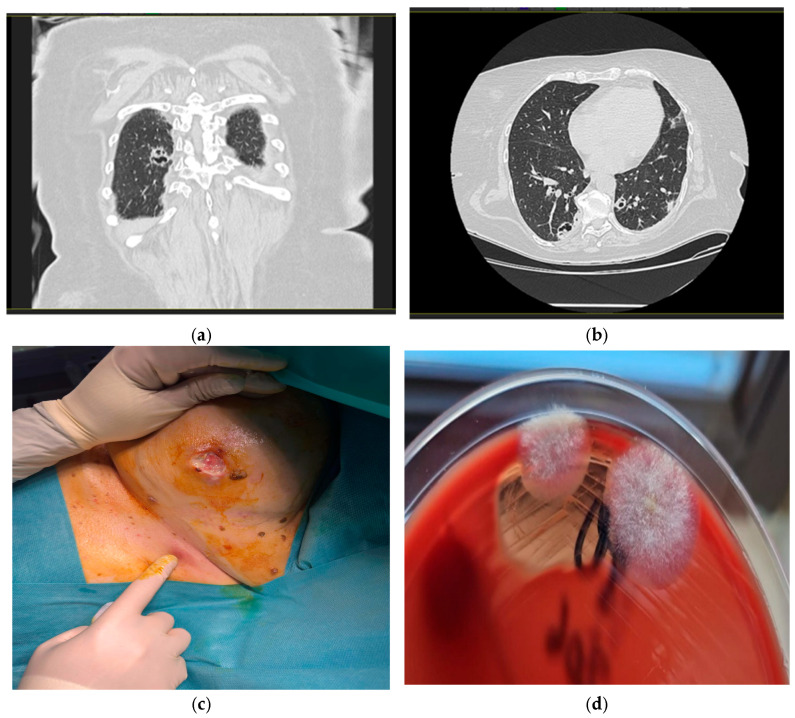
Radiologic and microbiologic features of cavitary lesions. (**a**) Coronal CT image showing a cavitary lesion in the right upper lobe of the lung; (**b**) axial CT image of a cavitary lesion in right lower pulmonary lobe; (**c**) fistulized nodular lesion in the right submammary region; (**d**) sample culturing on Columbia agar with ram blood showed the white and cottony colonies.

At six days post discharge, the patient was urgently readmitted to the Internal Medicine department, showing a serious general condition, pale skin, and erythematous nodular formations on the lower and upper limbs and bilaterally, in the region of the mammary glands. She was feverish (39 °C), but conscious, cooperative, and normoxic (SpO_2_ = 97%), with normal heart rate (89 bpm) and blood pressure (110/70 mm Hg). Pulmonary auscultation revealed crackles in both lungs. Leukopenia (below-normal leukocyte count), thrombocytopenia (low platelet count), SIRS, higher-than-normal BUN values, normochromic normocytic anemia (anemia with normal cell indices), hypoalbuminemia, and hypoproteinemia were also detected. A chest CT revealed multiple round-oval, well-demarcated, bilateral pulmonary cavities, more pronounced in the lower lobes, and especially on the right lobe (Figure 1a,b). This part exhibited irregular, anfractuous edges, some of which were conglomerated and presented nodular areas inside. The maximum dimensions of these nodules were 22/18 mm in the posterior segment of the right lower lobe.

Urinary, pharyngeal, nasal, and blood cultures were collected. Bronchoscopy was performed, with bronchial aspirate being collected and used for culture of common (commensal) microbiota, mycological examination, and testing for the presence of Koch’s Bacillus. Given the suspicion of a nosocomial infection secondary to ICU admission, empirical antibiotic therapy with carbapenem was administered, together with corticotherapy, PPIs, probiotics, analgesics, antioxidants, blood products (including a platelet concentrat), hepatoprotector agents, and loop diuretics. Microbiological culture from the bronchial aspirate revealed the presence of *Klebsiella pneumoniae* and *Providencia stuartii*. Antibiotic therapy was adjusted based on the antibiogram and included a dual regimen with a carbapenem (meropenem) and a polymyxin (colistin), with dosage adjustments made according to the patient’s glomerular filtration rate (GFR). Fungal culture from bronchial aspirate was negative.

The patient’s evolution was unfavorable: biological parameters (including systemic inflammatory markers) improved and BUN levels returned to normal, but there was an increase in size and fistulization of erythematous nodular elements on the lower and upper limbs and those located bilaterally from the mammary glands (Figure 1c). It was decided to request a minor plastic surgery involving incision and debridement of certain large lesions, collect tissue samples for fungal and bacterial culture, and perform a histopathological examination. These samples were inoculated onto blood agar, Sabouraud agar, and deoxycholate-citrate-lactose (DCL) agar according to the standard microbiological procedures, within one hour of collection. The presence of Gram-negative bacteria was identified on blood agar and DCL medium, and confirmed using Gram staining.

The cultures were examined and interpreted according to criteria for purulent secretion cultures. Bacterial isolates were subsequently inoculated for VITEK identification and antibiogram using VITEK^®^ 2 Compact antibiogram strips (BioMérieux, Inc., Hazelwood, MO, USA). The analysis was performed using the identification card (card type: GN) and antibiotic card (card type: AST-N204) for Gram-negative bacteria. Data were analyzed using the VITEK 2 software version 9 according to the manufacturer’s guidelines. Microbial identification was performed with Matrix-assisted laser desorption/ionization time-of-flight (MALDI-TOF) mass spectrometry (MS) on VITEK MS (BioMérieux, Inc., Hazelwood, MO, USA) as per the manufacturer’s instructions and internal laboratory standards. The Gram-negative bacterium was identified as *Klebsiella pneumoniae* spp.; its antibiotic sensitivity profile is given in Table 2. The recovered organism showed sensitivity to gentamicin and trimethoprim/sulfamethoxazole, but resistance to the other antibiotics tested.

Histopathological examination of samples—small fragments of mammary gland—revealed the presence of widespread lesions of fat necrosis accompanied by a rich, diffuse lymphoplasmacytic and polymorphonuclear inflammatory infiltrate; and vascular congestion with associated blood extravasations. Extensive colonization with septate hyphae, elongated and exhibiting regular progressive branching at approximately 45 degrees was observed at the margin of the lesions, most likely fungal infection with *Aspergillus*. Moreover, multiple fragments of mammary parenchyma presented acute suppurative inflammation processes in fibroadipose tissue, areas of fibrosis and fat necrosis, and hyphae (with the same features described above) located at the tissue margin.

Tissue specimens obtained during surgical debridement and allocated for mycological analysis were inoculated onto Columbia agar with 5% ram blood, MacConkey agar, Briliance UTI agar, mannitol salt agar, and Sabouraud agar with chloramphenicol 1.30 h post collection, and then incubated aerobically at 37 °C. We inoculated multiple culture media were inoculated to obtain a broad overview of potential pathogens, ensure optimal recovery of both fungal and bacterial organisms and allow exclusion of a possible mixed infection. Incubation was performed exclusively at this temperature since this value reflects the physiological conditions encountered by microorganisms in host tissues. A wet mount prepared following inoculation showed the absence of leukocytes and the presence of mycelial filaments. The Gram-stained smear demonstrated absence of polymorphonuclear cells and bacterial flora. In addition, two flat, fluffy colonies (1–2 cm in size) were found on Columbia agar with 5% ram blood at 48 h post-incubation (Figure 1d). All these findings were suggestive of a fungal etiology.

**Figure 2:**
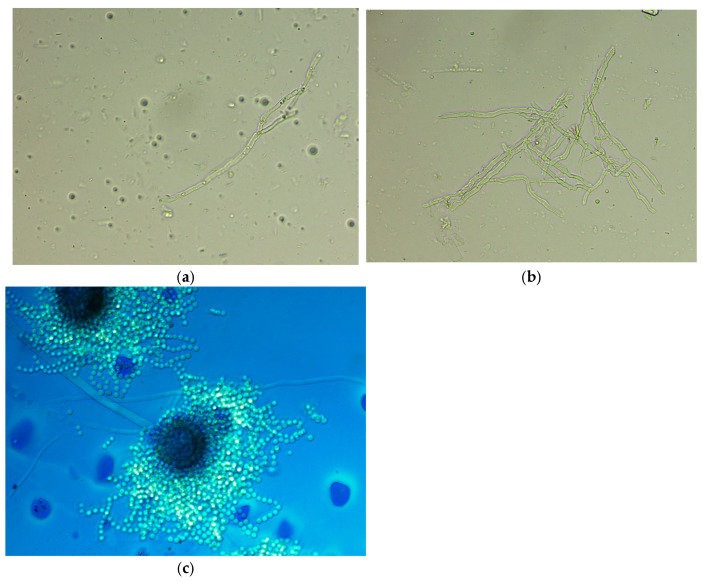
Bright-field microscopy (40× objective). (**a**) Native preparation showing an isolated fragment of septate hyphae consistent with *Aspergillus* spp.; (**b**) native preparation revealing a dense, branched network of septate hyphae characteristic of *Aspergillus* spp.; (**c**) Lactophenol cotton blue staining showing a mature *Aspergillus* spp. conidial head.

After performing a native preparation (Figure 2a,b) of the colonies, we identified the presence of mycelium and conidial heads. Lactophenol cotton blue staining (Figure 2c) revealed hyphae and aspergilliform heads with spherical vesicle; hyaline, finely echinulate conidiofor; and spherical, echinulate conidia. The laboratory report was issued, indicating with the presence of fungal colonies with microscopic and macroscopic appearance of hyaline septate filamentous fungi suggestive of the genus *Aspergillus*. Precise fungal species identification was performed using matrix-assisted laser desorption/ionization time-of-flight mass spectrometry (MALDI-TOF MS). In this case, the isolate was identified as *Aspergillus flavus* using the VITEK MS MALDI-TOF system, following the manufacturer’s standard protocol for filamentous fungi, which included an on-target extraction step with 70% formic acid to enhance protein spectrum quality. Fungal sensitivity against isavuconazole, itraconazole, and voriconazole was determined using the gradient E-test method. These antifungal agents were chosen based on documented species susceptibility reported in EUCAST v11.0 (2024). The results of this test are given in Table 3.

The patient followed treatment according to the antibiogram and antifungal chart, with aminoglycoside and voriconazole. The clinical course was stable, with improvement seen in clinical and biological parameters. However, lesions in the mammary glands progressed finally requiring bilateral mastectomy.

## 3. Discussion

The present case is unique in the medical literature as the first report to document the primary cutaneous infection of *A. flavus* occurring concurrently with *K. pneumonia*. The evidence accumulated to date on this topic is notably scant and limited to data derived from invasive pulmonary infections. Thus, Xu et al. described chronic aspergilloma and concomitant invasive pulmonary aspergillosis in a 62-year-old male, with *A. flavus* coinfecting with hypervirulent *K. pneumoniae* [6]. A similar clinical condition—severe pneumonia and pulmonary aspergillosis—was reported by Bhatia et al. in a 66-year-old male former smoker with chronic obstructive pulmonary disease (COPD) diagnosed with concurrent coinfection by *Aspergillus fumigatus* and *K. pneumoniae* [7]. Unlike these cases, the present infection manifested as extensive skin and mammary lesions.

We note that literature data on cutaneous infections with *A. flavus*, particularly when accompanied by *Klebsiella pneumoniae*, remain rare, with *A. fumigatus* being the most frequently implicated species in pulmonary infections. Moreover, such mixed infections typically affect the lungs and occur in immunocompromised patients, e.g., cases with COPD, diabetes, or prolonged corticosteroid use. In contrast, we studied a initially immunocompetent patient who only later developed opportunistic infections. Similarly to our findings, Xu et al. described a case of *A. flavus* and *K. pneumoniae* pulmonary coinfection in a critically ill patient with leukopenia, where metagenomic sequencing facilitated diagnosis [6]. In patients with COPD, Bhatia et al. reported coinfection with *Klebsiella pneumoniae* and *Aspergillus fumigatus* [7], whereas Das et al. described coinfection with *Pseudomonas aeruginosa* and *A. fumigatus* [8]; prolonged dual antimicrobial therapy led to recovery in both cases. In contrast, Luo et al. and Zhao et al. documented disseminated fungal–bacterial infections with poor outcomes, especially in elderly or diabetic patients [9,10]. Notably, our case is among the few to report extensive cutaneous involvement, likely stemming from hematogenous dissemination, and required surgical intervention (mastectomy) for source control despite appropriate antifungal and antibacterial therapy.

Cutaneous coinfection with *Klebsiella pneumoniae* (Gram-negative bacterium, frequently antibiotic resistant) and *Aspergillus flavus* (aflatoxin-producing fungus with both superficial and invasive potential) is clinically plausible, especially in immunocompromised patients or those with skin or necrotic lesions [4,5,11,12]. However, there was no evidence to suggest a compromised immune status, given the absence of documented prior hospitalizations and known comorbidities. Although cavitary lesions described on the CT scan performed prior to dermal extension were suggestive of fungal lung disease, the bronchial aspirate culture came out negative for fungi. This absence of detectable fungal growth supports a different dissemination pattern compared with classical pulmonary aspergillosis. In light of the present findings, we suggest that the cutaneous dissemination of this coinfection occurred via blood and secondary to the pulmonary infection. Multiple converging lines of evidence—radiologic signs of primary pulmonary involvement, the temporal sequence of lesion appearance, the multifocal and bilateral distribution of nodules, histopathologic demonstration of deep angioinvasive fungal elements, and the absence of any local cutaneous inoculation—strongly support this hypothesis.

The medical literature provides several potential explanations for the aforementioned absence of fungal growth in the presence of *Klebsiella pneumoniae.* First, bronchial aspirate has limited sensitivity for fungal pathogens [13,14]. Second, cavitary lesions may also result from the concomitant *Klebsiella pneumoniae* infection [9]. A third and particularly credible mechanism is fungal inhibition in the presence of *Klebsiella.* In fact, in vitro evidence shows that *Klebsiella pneumoniae* can suppress spore germination and hyphal development in several *Aspergillus* species, including *Aspergillus flavus,* with primary cutaneous bacterial infection masking the fungal component in cultures. This direct, contact-mediated and reversible inhibition requires the presence of live, actively growing bacteria. During the interaction with *Klebsiella pneumoniae*, *Aspergillus* adopts a “dormant” state during which the genes involved in hyphal development are downregulated, thus providing the minimum energy necessary for survival [15]. Moreover, the fungus enters a defensive state characterized by wall remodeling and strengthening and increased expression of genes involves in these processes and oxidative stress response [13]. Similar mechanisms have been already been described in other fungi, with activation of cell wall salvage pathways as a rescue mechanism in response to cell wall damage stress [16,17,18]. Therefore, understanding the mechanisms involved in bacterial–fungal interactions is essential for the development of appropriate diagnostic and therapeutic approaches in these coinfections.

While biofilm formation per se can lead to resistance to antimicrobial treatment due to limited antibiotic penetration into the biofilm [19,20,21], the additional effects of microbial interactions within mixed biofilms that impair the defense mechanisms of individual pathogens may further enhance resistance to therapy. In our case, the co-infecting *K. pneumoniae* was an extensively drug-resistant, hypervirulent strain, and the *A. flavus* was susceptible only to azoles (voriconazole/itraconazole/isavuconazol). It is highly likely that the co-presence of these pathogens amplified their virulence, with lesions progressing until bilateral mastectomy was required despite appropriate antibiotics and antifungals.

The present case report suggests that separate sampling for bacterial culture, fungal culture, and histopathological examination is the best approach in the context of suspected fungal infection. Moreover, histopathological examination with special stains (Periodic Acid–Schiff, Gomori–Grocott) to detect intratisular fungal hyphae/elements is recommended even if fungal culture is negative [22], as was demonstrated in our patient. In contrast, molecular tests (PCR) and/or antigen detection are useful in systemic aspergillosis but lack sensitivity in cutaneous infections [23]. Taken together, these considerations highlight the importance of correct sampling and histopathological confirmation for diagnosing cutaneous fungal infections.

An important point concerns the possibility of missed diagnosis during the initial hospitalization. We found no clinical signs suggestive of fungal infection during the initial admission, with thoracic imaging findings being consistent with a bacterial septic process rather than a fungal etiology. Bronchial aspirate cultures were also negative for fungi and no radiological hallmarks (e.g., cavitations, nodules) prompting suspicion for invasive aspergillosis existed. It was only upon readmission—marked by new-onset cutaneous nodules with necrosis and histological evidence of angioinvasion—that targeted mycological investigations were pursued. This progression highlights the diagnostic complexity in cases with a secondary fungal infection following immune dysregulation due to sepsis or intensive care interventions. Our experience underscores the need for vigilant re-evaluation and broadened differential diagnosis in patients with atypical evolution, particularly when standard antimicrobial therapy fails to halt disease progression.

As a general principle of treatment, medical professionals should treat both bacterial and fungal infections with targeted therapies based on susceptibility testing in conjunction with local surgical management of the lesions (e.g., debridement, drainage). According to current antibiotic therapy guidelines, the standard therapy for severe/nosocomial skin infections with *Klebsiella pneumoniae* involves the use of third generation cephalosporins, aminoglycosides, or fluroquinolones in the absence of extended spectrum beta-lactamase resistance (ESBL) [24,25]. The administration of carbapenems is recommended for ESBL-producing strains, whereas cetfazidime–avibactam, meropenem–vabrobactam, or imipenem–cilastatin–relebactam are typically used for carbapenemase-producing strains [26,27]. Voriconazole showed here the lowest MIC value, supporting its selection as antifungal drug. This is in line with evidence-based data recommending its use as first-choice antifungal agent for most invasive forms of aspergillosis, typically at a range between 1 and 4 mg/L to prevent both treatment failure and toxicity. In this context, therapeutic drug monitoring plays an important role in optimizing voriconazole therapy [26,27]. In the case of localized cutaneous forms, the best results are provided by surgical debridement in association with antifungal administration [26,27,28]. Given the lack of validated protocols for topical/local therapies, systemic antifungal treatment remains the optimal approach in the case of cutaneous *Aspergillus* lesions.

This study reveals the diagnostic complexity and clinical severity of rare cutaneous coinfection involving *Aspergillus flavus* and *Klebsiella pneumoniae* in a patient immunocompetent at baseline who developed secondary immunosuppression following septic shock. Such overlapping infections require careful clinical monitoring since opportunistic fungal infections can occur even in patients without classic risk factors and angioinvasive fungal lesions often present with delayed onset. Repeated diagnostic reassessment is recommended in cases when infection persists or progresses despite targeted antibacterial therapy. Early surgical intervention, comprehensive mycological investigation, and a multidisciplinary approach were essential in achieving a favorable outcome. This case also contributes to the limited literature on fungal–bacterial cutaneous coinfections, provides new insights into their diagnostic and therapeutic challenges.

## Figures and Tables

**Table 1:** Antibiotic susceptibility for *Staphylococcus epidermidis*.

Antibiotic	MIC (mg/L)	Interpretation
Oxacilin	>2	R
Gentamicin	>8	R
Ciprofloxacin	>4	R
Moxifloxacin	>4	R
Erythromycin	1	S
Clyndamicin	>4	R
Linezolid	>4	R
Teicoplanin	16	R
Vancomycin	1	S
Tetracycline	4	R
Tigecycline	0.25	S
Fusidic acid	>16	R
Rifampicin	>16	R
Trimethoprim/Sulfamethaxozole	80	R

MIC, minimal inhibitory concentration; mg/L, milligrams per liter; R, antibiotic resistance; S, antibiotic sensitivity.

**Table 2:** Antibiotic susceptibility for *Klebsiella pneumoniae*.

Antibiotic	MIC (mg/L)	Interpretation
Termocillin	>16	R
Ampicillin	>16	R
Amoxicillin/Clavulanic acid	>32	R
Ampicillin/sulbactam	>16	R
Piperacillin/Tazobactam	>64	R
Cefuroxime	>32	R
Cefotaxime	>32	R
Cetriaxone	>32	R
Ceftazidime/Avibactam	>8	R
Ceftolozane/Tazobactam	>16	R
Cefepime	>16	R
Aztreonam	>32	R
Ertapenem	>4	R
Imipenem	>8	R
Meropenem	>8	R
Imepenem/Relebactam	>8	R
Gentamicin	<1	S
Tobramycin	>8	R
Ciprofloxacin	>2	R
Levofloxacin	>4	R
Moxifloxacin	>4	R
Colistin	>4	R
Trimethoprim/Sulfamethoxazole	40	S

MIC, minimal inhibitory concentration; mg/L, milligrams per liter; R, antibiotic resistance; S, antibiotic sensitivity.

**Table 3:** Antifungal susceptibility obtained for *Aspergillus flavus*.

Antifungic	MIC (mg/L)	Interpretation
Isavuconazole	0.50	AS
Itraconazole	1	AS
Voriconazole	0.38	AS

MIC, minimal inhibitory concentration; mg/L, milligrams per liter; AS, antifungal sensitivity.

## Data Availability

The original contributions presented in this study are included in the article. Further inquiries can be directed to the corresponding author.

## Abbreviations

The following abbreviations are used in this manuscript:

AIDS	Acquired Immunodeficiency Syndrome
AS	Antifungal Sensitivity
BUN	Blood Urea Nitrogen
COPD	Chronic Obstructive Pulmonary Disease
CT	Computed Tomography
DCL	Deoxycholate-Citrate-Lactose Agar
ESBL	Extended-Spectrum β-Lactamase
EUCAST	European Committee on Antimicrobial Susceptibility Testing
GFR	Glomerular Filtration Rate
ICU	Intensive Care Unit
*K. pneumoniae*	*Klebsiella pneumoniae*
MALDI-TOF MS	Matrix-Assisted Laser Desorption/Ionization Time-of-Flight Mass Spectrometry
MIC	Minimum Inhibitory Concentration
PPI	Proton Pump Inhibitor
R	Antibiotic Resistance
S	Antibiotic Sensitivity
SIRS	Systemic Inflammatory Response Syndrome
spp.	Species (plural)
UTI	Urinary Tract Infection
XDR	Extensively Drug-Resistant
